# Psychological Climate for Caring and Work Outcomes: A Virtuous Cycle

**DOI:** 10.3390/ijerph17197035

**Published:** 2020-09-25

**Authors:** Dorota Weziak-Bialowolska, Piotr Bialowolski, Carlued Leon, Tamar Koosed, Eileen McNeely

**Affiliations:** 1 Sustainability and Health Initiative (SHINE), Department of Environmental Health, Harvard T.H. Chan School of Public Health, Boston, MA 02115, USA; pbialowolski@hsph.harvard.edu (P.B.); emcneely@hsph.harvard.edu (E.M.); 2 Manaus, LLC, Los Angeles, CA 91436, USA; cleon@manausconsulting.com (C.L.); tamar@manausconsulting.com (T.K.)

**Keywords:** psychological climate, climate for caring, job satisfaction, work engagement, self-reported work outcomes

## Abstract

The current literature’s focus on unidirectional effects of psychological and organizational climates at work on work outcomes fails to capture the full relationship between these factors. This article examines whether a psychological climate for caring contributes to specific work outcomes and investigates whether work outcomes support the climate for caring, creating a feedback loop. Results confirm a bi-directional, temporal association between perceived climate for caring and two of the four explored work outcomes: self-reported productivity and self-reported work quality. The effect of a perceived caring climate on these work outcomes was stronger than the effect in the opposite direction. The perception that the work climate was caring was also found to affect work engagement, but the reverse relationship was not identified. We did not find any evidence for a link between job satisfaction and a climate for caring at work in either direction.

## 1. Introduction

For many decades research and practice strived to improve organizational culture and climate as a means to boost organizational effectiveness. More recently, the pursuit of a more caring psychological climate that makes employees feel cared for, treated fairly, and with respect has gained attention among managers and academics as a way to improve employee motivation and boost business outcomes [1]. However, the literature still lacks empirical evidence on the full effects and feedback loops that take hold between positive or negative work climates and work outcomes and attitudes. With empirical evidence, this paper sheds new light on this important relationship.

Psychological climate is defined as the meaning attached by employees to the events, policies, practices, and procedures in an organization as well as employees’ identification of the behaviors perceived as rewarded, supported, and expected, which—being a salient social contextual factor—helps employees succeed in the work environment [2,3]. Psychological climate for caring, in particular, focuses on employees’ perceptions of management care about the workforce, and management priorities regarding trust, respect, fairness, caring atmosphere, and safety in the workplace. Organizational climate is a shared perception or a shared meaning attached to the byproduct of the core values, beliefs, and assumptions of an organization [4,5,6,7], that is, it is an attribute of a group or an organization and not of an individual employee. The climate (either psychological or organizational), contrary to culture, is behaviorally oriented [8] and it has a narrow scope, focusing often on specific goals or internal processes [2]. Examples of specific goal-oriented climates include climates for safety [9,10] and for innovation [11,12]. Examples of internal process-oriented climates include climates for trust [13], for justice [14], for ethical work [15,16], or for caring [17].

The impact of psychological climate on specific work outcomes (such as job satisfaction, work engagement, self-assessed productivity, and performance) is well documented in the literature [18,19,20,21,22,23]. Considerably fewer studies, however, explore the reverse effect—the impact of work outcomes on psychological climate. Those that do, rely on cross-sectional data and thus provide a limited base for assessment of bi-directional links. In this paper we contribute to these gaps in the research.

### 1.1. Motivation and Research Questions

In this study, we focus on the psychological climate—the perception employees have of their work environment and its impact on their own work outcomes. Additionally, following recent trends and approaches to examine the “climates for specific, work-related aspects” [3], we focus on employees’ perceptions of the psychological climate for caring and seek to establish its role for work outcomes. Simultaneously, we try to establish the role of work outcomes for building the psychological climate for caring. To this end, we test the relationship between perception of psychological climate for caring and work outcomes in both directions. We use three-year longitudinal data from apparel workers in Mexico to answer the following research questions:

(i). What is the effect of workers’ perceptions of the climate for caring on subsequent job satisfaction, work engagement, self-reported productivity, and self-reported work quality?
(ii). What is the effect of job satisfaction, work engagement, self-reported productivity, and self-reported work quality on workers’ subsequent perceptions of the psychological climate for caring?
(iii). If both temporal associations are present, is the effect of perception of climate for caring on work outcomes weaker or stronger than the effect in the reverse relationship?

Our approach to these questions is novel and adds to the literature. It provides stronger evidence for the traditionally explored relationship that psychological climate in general and climate for caring in particular impact work outcomes by using three-wave longitudinal data—as cross-sectional estimates usually misestimate the existence of or overestimate the strength of these relationships [24,25]. Our approach also provides evidence for the reverse temporal association between work outcomes and the perception of climate for caring, demonstrating a feedback loop rarely explored in the literature. Our findings provide new arguments for the creation and nurturing of a positive, caring culture for an organizational success.

### 1.2. Conceptual Framework and Research Hypotheses

Our study builds on two theoretical approaches: the non-recursive model of causal relationship between job perception domain of psychological climate and job satisfaction proposed by James and Tetrick [26], and the job demands-resources model of Bakker and Demerouti [27,28,29]. Although these approaches use different terminology, both focus on the relationships between job perception and/or job resources and work outcomes. Both models also assume a reciprocal relationship between these elements. However, the models differ in their focus. James and Tetrick [26] focus on psychological climate and its effect on work outcomes, while Bakker and Demerouti [27,28,29] explore how work outcomes are affected by job resources and job demands. Since psychological climate is classified as a job resource [30], both models are complementary for our research and using both is adequate for our investigation of the reciprocal relationship between psychological climate and work outcomes. Both are presented below.

The non-recursive model of causal relationship between job perception domain of psychological climate and job satisfaction was proposed by James and Tetrick [26]. It was further validated by James and James [31] and corroborated by Mathieu et al. [32]. This model assumes that the work environment and its psychological climate in particular can serve as a basis for the interpretation of and a guide for workplace behaviors. This stems from the assumption that psychological climate influences cognitive and affective states, such as job satisfaction. It also implies that cognitive and affective states may be antecedents to job perceptions and psychological climate. In other words, the model presents the rationale that employees develop a generalized reaction to the work environment that is subsequently reflected in their perception of job characteristics and their job attitudes. Once the perceptions of job characteristics and attitudes are formulated, they may feedback and modify affective responses to the work environment. As such, the model suggests that psychological climate and work outcomes are reciprocally related, positively reinforcing each other.

The original model by James and Tetrick [26] examines the relationship between three main elements: (1) job satisfaction, (2) psychological climate, and (3) job attributes and work-group structures (which include job complexity, job pressure, and specialization of structure, among others). Two alternative specifications for estimating reciprocal relationships between the three identified elements were presented. The specifications differ with respect to the roles of the major elements. In the first specification (the postcognitive-nonrecursive model), it is assumed that psychological climate mediates the relationship between job attributes and job satisfaction, while the relationship between job satisfaction and psychological climate is reciprocal. In the second specification (the precognitive-nonrecursive model), job satisfaction is assumed to mediate the relationship between job attributes and psychological climate, while the relationship between job satisfaction and psychological climate is still reciprocal.

**Figure 1:**
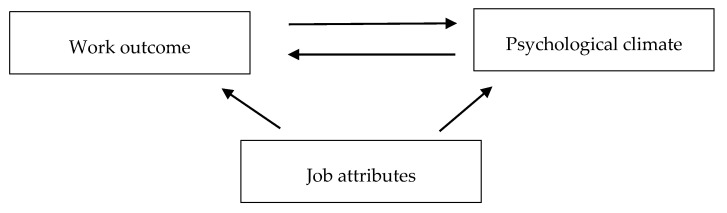
Model relating psychological climate and individual work outcomes.

In our approach, we extend this model by employing, beyond job satisfaction, three additional work outcomes. We also tested the nonrecursive part of the model. Although job attributes were accounted for as control variables (henceforth also referred to as job characteristics) in our approach, the precognitive/postcognitive paths were not tested as this was beyond the scope of the study. Our model is presented in Figure 1.

Our examination of associations between psychological climate and specific work outcomes, such as job satisfaction, work engagement, and performance also benefited from the theoretical considerations of the job demands-resources model of Bakker and Demerouti [27,28,29]. The model shows that job demands (e.g., workload or time pressure) moderate the relationship between job resources (e.g., feedback, autonomy, social support, and organizational climate) and positive, well-being-related work outcomes. The assumption is that both job and personal resources can favorably affect work outcomes, especially work engagement. This positive effect is supposed to be further strengthened by moderate and high job demands. However, a meta-analytical study showed that correctness of this assumption is highly dependent on the nature of the demand (i.e., challenges vs. hinderances) [30]. Finally, the model assumes that there is a loop between work engagement and job resources, implying that employees with high work engagement can create their own job resources, which in turn can stimulate engagement and create a positive feedback loop [27]. Although the originally developed model focused on the relationship between job resources and work engagement, other positive work outcomes considered by Bakker and Demerouti comprised performance, professional efficacy, and organizational commitment and dedication, among others [33,34]. Some scholars also applied the model to predict job satisfaction [35,36].

Clear distinction has been made between the organizational and psychological climate. The former is defined as a property of an organization and the latter as a property of its employees [3,37,38,39]. Consequently, psychological climate is an individual level perception of organizational climate and reflects the attitudes, meaning, and significance expressed by employees, while organizational climate is viewed as the outcome emerging from aggregation of individuals’ shared psychological climates [3,8].

Five dimensions of psychological climate perceptions have been identified [39,40]: (1) role stress and lack of harmony, (2) job challenge and autonomy, (3) leadership facilitation and support, (4) work-group cooperation, friendliness, and warmth, and (5) organizational and subsystem attributes. The meta-analysis conducted by Parker et al. [40] showed that there is a positive association between dimensions of psychological climate, work attitudes, and employee performance. Our climate for caring corresponds to the fifth dimension—the organizational and subsystem attributes—of the psychological climate as defined by Jones and James [39].

There is consensus that psychological and organizational climates play an important and positive role in company performance [17,20,22,41,42,43,44,45,46] and create capacity for sustained competitive advantage [47,48,49,50,51,52]. Both concepts have been used to explain employees’ motivational and affective reactions to change in the work environment [40]. At the employee level, psychological climate—sometimes also referred to as organizational climate at individual level—has been linked to productivity and work quality. Higher levels of these work outcomes were observed among employees experiencing better work climate [53,54,55]. There is also evidence that psychological climate in general, and climate for caring in particular, are positively correlated with job satisfaction [17,20,21,39,44,45,56], work engagement [57,58], and job performance [17]. Conversely, perceptions of negative working climate were linked to employee theft and sick-day abuse [59,60,61]. The theoretical considerations presented above and the empirical findings previously explored in the literature allowed us to hypothesize that the climate for caring will favorably affect individual work outcomes such as job satisfaction, work engagement, quality of work, and productivity (Hypothese H1).

The bi-directional relationship between psychological climate and work outcomes has been acknowledged in the organizational literature [26] but has not been widely explored. Siehl and Martin [24] proposed two possible explanations for the reverse direction of causality. The more cynical one states that a good financial performance may lead to the promotion of a desirable organizational image to explain unusually high or low profits. The second explanation states that high levels of performance provide resources to fund the promotion and endorsement of organizational values such as safety, respect, fairness, and caring for people.

The empirical evidence on work outcomes as antecedents of either psychological or organizational climate is scarce. In the service industry, studies have shown that service quality may significantly positively influence service climate, but the reverse relationship was not ascertained [62]. Similarly, no significant temporal impact of productivity on the organizational climate was evidenced [63]. Nevertheless, this evidence, as well as assumptions of the two theoretical models presented above, led us to formulate the hypothesis that individual work outcomes will positively affect the climate for caring (Hypothese H2).

Positive reciprocal relationships between psychological climate (expressed as job perceptions) and job satisfaction were evidenced but only using cross-sectional data [26,31,32]. Despite the assertion of James et al. [3] that the reciprocal causation model of the job perception/psychological climate—job satisfaction relationship is empirically supported, we argue that the present evidence is insufficient because it relies on cross-sectional data and evidence of a correlational nature. Consequently, we test the hypothesis that there is a reciprocal favorable relationship between climate for caring and individual work outcomes such as job satisfaction, work engagement, quality of work, and productivity (Hypothese H3).

## 2. Materials and Methods

### 2.1. Data Source and Sample Size

The Worker Well-Being Survey (WWBS) provided data for the study. The WWBS is a tool that was developed to track well-being among garment workers in the global supply chains of an international brand. The tool was applied in the apparel factories in Cambodia, China, Mexico, and Sri Lanka that expressed interest in worker well-being programs provided by the brand and had successfully passed external compliance audits with environmental measurement tools designed to examine and measure organizational non-financial performance. The survey development process is presented elsewhere [64,65].

Data used were collected from 495 apparel workers in Mexico who participated in three annual waves of the WWBS. The first wave of the WWBS was administered in February 2017, the second in March 2018, and the third in March 2019. Table 1 presents the descriptive statistics for the longitudinal sample.

In total, 2355 workers (51.7% of the total workforce) were invited to participate in the first wave of the survey and 2278 took the survey (response rate of 96.1%). In the second wave 2486 workers took part in the survey (67.4% of the total workforce) and 2723 participated in the third wave (70.8% of the workforce). However, due to the high turnover rates, the retention rate from wave 1 through wave 2 and to wave 3 was 22.5%. The final panel dataset with individuals who responded in all 3 waves comprised 495 employees.

The WWBS was administered on tablets in private areas separate from employees’ workstations. To minimize disruptions in factory operations, workers were surveyed in batches (e.g., one production line at a time). Eighty high-quality t-shirts with logos of either a global apparel brand or an Ivy League university, as well as three smartphones, were offered as incentives to randomly selected participants at each data collection wave. Participation was voluntary and confidential, as workers could decline to participate within the tablet application without their supervisor or factory management knowing.

All workers signed an informed consent. The study was reviewed and approved by the Harvard Longwood Campus Institutional Review Board (IRB14-3500). Researchers shared with workers and management only aggregate survey results.

### 2.2. Measures and Variable Specification

#### 2.2.1. Climate for Caring

To operationalize the perception of psychological climate for caring, the caring climate scale proposed by McNeely et al. [66] was used. This 6-item instrument measures employees’ opinions on management care about the workforce, and management priorities in the realms of trust, respect, fairness, caring atmosphere, and safety in the workplace. The instrument is intentionally concise to enable widespread use and cognition.

While we are aware that the measurement of psychological climate is complex and usually conducted using multi-item scales due to the multi-dimensionality of the concept [3,39,67], we argue that long instruments allow for less flexibility, especially when used in workplace studies where many concepts are measured at the same time. A similar argument has been recently made by other scholars regarding well-being measurement [68,69]. Additionally, by focusing specifically on the climate for caring, we follow the approach of psychological climate with a specific strategic focus [7]. Finally, given that most organizational and psychological climate instruments have not been validated [8] or present questionable psychometric properties [8], we selected an instrument that has been already proven effective in measuring perception of apparel factory workers. McNeely at al.’s instrument has been tested on a sample of over 15,000 garment workers in China, Cambodia, Mexico, Poland, Sri Lanka, and the United States and on over 5500 office and manufacturing employees of two Fortune 500 manufacturing companies in the United States [54,66].

Six indicators of perception of psychological climate for caring were considered: (1) Management truly cares about the employees; (2) Safety is a high priority in my workplace; (3) Employees feel respected at work; (4) Employees trust management; (5) Employees feel they are treated fairly; and (6) Management helps me deal with stressful situations at work [66]. Respondents were asked to assess their level of agreement with these statements using a 4-point Likert scale (1 = strongly disagree; 2 = disagree; 3 = agree; 4 = strongly agree). These six indicators were subsequently used to construct a composite measure of perception of the psychological climate for caring, with higher values indicating better climate. The psychological climate scale was tested using confirmatory factor analysis (CFA) and demonstrated sufficient psychometric properties in our sample (CFI = 0.979, TLI = 0.966, SRMR = 0.027, RMSEA = 0.080; based on the third wave of data). Cronbach’s alpha coefficient was 0.895. Association between the scale and work outcomes such as job satisfaction, work engagement, number of days with limited ability to perform work tasks and positive affect at work was positive as expected, providing evidence for the criterion validity.

#### 2.2.2. Work Outcomes

Four work outcomes were selected for the analysis: self-reported productivity, self-reported work quality, job satisfaction, and work engagement. We relied on empirically proven indicators and scales for measuring them.

To measure a perception of productivity, employees were asked to self-assess the statement “Employees are productive and engaged” using a 4-point Likert scale (1 = strongly disagree; 2 = disagree; 3 = agree; 4 = strongly agree). To measure work quality, workers responded the following question “What do you think about the quality of work of your coworkers in jobs similar to yours?” using a 11-point scale (0 = Poor quality, 10 = Excellent quality). This is a modified version of the question available in Cammann et al. [70] that has been also proven to be effective in the workplace settings [64]. This indirect question was used to account for social desirability and employs a projective technique to evaluate performance of a similar individual acting in a particular situation [71]. It has been empirically shown to provide a valid and reliable assessment of the problem at hand [72,73]. Job satisfaction was measured using a single question: “Overall, how satisfied (content) are you with your job at this factory?” with workers using a 11-point scale to answer the question (0 = Not at all satisfied, 10 = Extremely satisfied) [74,75,76,77]. To measure work engagement, we used the shortened version of the Utrecht Work Engagement Scale-9. This 9-item instrument has well established psychometric properties (the median Cronbach’s alpha amounted to 0.92 across all countries in which the scale was validated; the one-factor model of UWES-9 fit to the data reasonably well with the standard fit indices meeting the criterion of 0.90) [78,79].

Descriptive statistics and correlations between perception of psychological climate for caring and work outcomes are presented in Table 2.

#### 2.2.3. Control Variables

Research shows that job attitudes differ depending on demographics such as gender, age, marital status, education, and job tenure [80,81,82,83,84,85,86,87,88,89,90,91,92]. Similarly, taking care of an elder has a detrimental effect on job satisfaction and other job attitudes [93,94]. Additionally, there are theoretical foundations and empirical evidence that job attributes, such as psychological and physical job demands, job control, and learning opportunities correlate with job attitudes [3,95,96,97,98].

Work–family conflict has been theoretically and empirically shown to have a detrimental impact on job satisfaction [83,99], work engagement [100,101], ability to work [102], turnover [103,104], and intent of turnover [105]. At the same time, organization-led work-life balance programs have been empirically proven to improve productivity and other business outcomes [106,107].

Finally, work in an apparel factory is typically associated with considerable physical burdens and requires prolonged unnatural body positions, repetitive movements, and physical hazards, such as insufficient lighting, heat and cold stress, humidity, poor air quality and circulation, and noise [108,109]. These factors may significantly influence well-being, productivity, and performance [110,111,112,113].

Based on this theoretical and empirical evidence, our analysis controlled for the following variables related to both work outcomes and climate for caring: (1) demographic variables (gender, age, marital status, education, taking care for an elder, and having children below 18 at home); (2) job characteristics (job tenure, psychological and physical job demand, job control, learning opportunities, and physical working conditions); and (3) work-family conflict to make the assumption of no confounding for the effect of exposure on outcome conditional on a set of covariates as plausible as possible [114].

#### 2.2.4. Statistical Analysis

The two directions of the relationship between perception of psychological climate for caring and work outcomes (i.e., how perception of caring climate influences work outcomes and vice versa) were explored using a lagged linear regression model. The relationship was modeled as follows:

(1) $$WO_{i,k}\left(T=3\right)=\alpha_{0}+\alpha_{1}CC_{i}\left(T=2\right)+\alpha_{2}X_{i}\left(T=1\right)+\alpha_{3}CC_{i}\left(T=1\right)+\alpha_{4}WO_{i,k}\left(T=1\right)+\eta_{i,k},\;i=1,\ldots ,N,\;k=1,\ldots ,4.$$

(2) $$CC_{i}\left(T=3\right)=\beta_{0}+\beta_{1}WO_{i,k}\left(T=2\right)+\beta_{2}X_{i}\left(T=1\right)+\beta_{3}CC_{i}\left(T=1\right)+\beta_{4}WO_{i,k}\left(T=1\right)+\varepsilon_{i,k},\;i=1,\ldots ,N,\;k=1,\ldots ,4.$$

where subscript *i* represents an individual, the variable *WO* indicates one out of four work outcomes (*k* = 1,…,4), and *CC* is the perception of psychological climate for caring. *X* is a vector of control variables. *α*_1_ and *α*_3_ reflect the effects of the perception of the psychological climate for caring on a work outcome at *T* = 2 and *T* = 1, respectively. *α*_2_ shows the association between control variables and the work outcome and *α*_4_–between a work outcome at *T* = 1 and *T* = 3. *β*_1_ and *β*_4_ show the effects of a work outcome on the perception of caring climate at *T* = 2 and *T* = 1, respectively. *β*_2_ shows the association between control variables and the perception of psychological climate for caring and *β*_3_–between the perception of caring climate at *T* = 1 and *T* = 3. *α*_0_ and *β*_0_ are intercept terms and *η* and *ε* are disturbance terms. *T* = 1, *T* = 2 and *T* = 3 indicate the wave of data collection.

This specification allows us to control for the baseline outcome (at *T* = 1), covariates prior to exposure (at *T* = 1), and prior values of the exposure variable itself (at *T* = 1). Controlling for baseline outcome helps mitigate the risk of reverse causation [114,115]. Controlling for covariates prior to exposure ensures that they are confounders and not mediators. Controlling for the value of the exposure in the prior wave of data (1) limits the risk of reverse causation and of unmeasured confounding and (2) facilitates interpretation of the effect estimates as a change in the exposure [114].

This methodological approach to establish relationships has been successfully adopted in the fields of well-being in work life [25], households’ financial behaviors [116], religiosity [117,118], social psychiatry [115,119], and epidemiology [120,121], among others.

Robustness of results was supported (1) by using three waves of data and thus controlling for reverse causation; (2) by using multiple imputations to account for the potential bias resulting from excluding observations in the complete case scenario; and (3) through the design of the study’s procedure to account for the common method bias [122].

For missing observations, we used 10 samples with predicted values following the multiple imputation logic. With multiply imputed data, we acknowledge that information about the missing variable is not deterministic and that for workers who are not reporting any response for a particular question, only a distribution of answers can be inferred from the data. Individuals who provided an answer were given the same response across all the samples. In contrast, individuals whose value was imputed were assigned in each sample a randomly drawn value from the predicted distribution. We then estimated the regression models (1) and (2) based on each of the 10 imputations and combined them using Rubin’s rule [123]. According to Rubin’s formula, the final set of regression coefficients for a regression model is calculated based on 10 estimates from each imputation and their respective variances. The point estimate for the final coefficient is a simple average of estimates ${\hat{\beta }}_{i}=\frac{\sum_{n=1}^{10}{\hat{\beta }}_{i,n}}{10}$, where ${\hat{\beta }}_{i,n}$ is *n*-th coefficient from the set of 10 coefficients estimated for each imputation *n* = 1, …, 10. If ${\hat{V}}_{i,n}$ represents variance of the estimate ${\hat{\beta }}_{i,n}$ in imputation *n*, then the formula for the variance of is obtained according to the following rule:

$${\hat{V}}_{i}=\frac{\sum_{n=1}^{10}{\hat{V}}_{i,n}}{10}+\frac{11}{9}\sum_{n=1}^{10}{\left({\hat{\beta }}_{i}-{\hat{\beta }}_{i,n}\right)}^{2}$$

The design of the study’s procedure accounted for the common method bias [122]. Specifically, predictor and outcome variables were located in different sections of the questionnaire (i.e., they were separated). Different response scales, e.g., 4-point Likert scales, number of days, intensity scales, 0–10 Likert type scales (see Table 2), with different scale endpoints and different labeling were also used to methodologically separate the variables.

Analyses were performed using Stata 15.

## 3. Results

We found evidence for the bi-directional relationship between perception of psychological climate for caring and two of the four work outcomes explored: self-reported productivity and self-reported work quality (Table 3). While the association ran in both directions, the impact of perceived climate for caring on work outcomes was slightly stronger. These results suggest two separate feedback loops between (1) productivity and perception of climate for caring and (2) work quality and climate for caring. They also corroborate hypotheses H1, H2, and H3 for these two work outcomes.

The largest effect size was observed for the temporal association between self-reported productivity and the perception of a climate for caring, in both directions (β = 0.19, *p* = 0.001 for the effect of climate for caring on the subsequent self-reported productivity and β = 0.17, *p* = 0.000 for the effect of self-reported productivity on the subsequent climate for caring). The positive link between the perception of psychological climate for caring and self-reported work quality was weaker in both directions (β = 0.14, *p* = 0.008 for the psychological climate’s effect on subsequent self-reported work quality and β = 0.11, *p* = 0.024 for the link in the opposite direction).

We have also found evidence for a positive effect of perceived psychological climate on subsequent work engagement (β = 0.16, *p* = 0.001), but no reverse effect was observed—the relationship was deemed unidirectional. This implies that only hypothesis H1 was confirmed for work engagement. In the case of job satisfaction, we found no evidence that this work outcome is related to either subsequent or prior perceived psychological climate for caring. Consequently, no support for the tested hypotheses was found.

Three of the four measured work outcomes were found to be associated with prior psychological climate for caring, while two of the four work outcomes were associated with the subsequent psychological climate for caring. This suggests that the perception of the psychological climate does not interact in the same way with all work outcomes. The size of the effect of psychological climate for caring on work outcomes was larger than in the reverse relationship. Therefore, while some bi-directional relationship between work outcomes and psychological climate exists, leading to a positive feedback loop, the relationship in our longitudinal dataset is larger and more prevalent in the direction from psychological climate for caring to work outcomes.

## 4. Discussion

By providing empirical evidence on the existence and directionality of the relationship between the perception of psychological climate for caring and selected work outcome variables, we argue that the established role of psychological climate as a bedrock for business performance and sustained competitive advantage is correct but incomplete. By using three-wave longitudinal data and exploring both directions of the relationship between work outcomes and the perception of psychological climate for caring, we found that work outcomes, such as self-reported productivity and self-reported work quality, are not only affected by psychological climate but also can be instrumental for its accumulation, with the relationship potentially functioning as a virtuous cycle. These results, therefore, corroborate hypotheses H1, H2, and H3 for these two work outcomes.

Similar to Schneider et al.’s [62] hypothesis on the role of organizational climate, but contrary to their results, we found that the psychological climate for caring is consequential to self-reported work quality, but the magnitude of the effect is higher from climate to self-reported work quality. Analysis of the relationship between customer perception of service quality and service climate by Schneider et al. [62] revealed that the path from service quality to climate for service was significant, while the reverse path was not, with some evidence of equal magnitude of the two paths. Differences in modeling strategies—despite both being appropriate—may have contributed to these differences. The target respondents also differed in their study, with our analysis concerned with apparel factory workers and theirs with service industry workers.

Contrary to van de Voorde et al. [63], who found no significant temporal impact of productivity on the organizational climate, our results show that higher self-reported productivity may lead to improved perception of the psychological climate in the following period. The difference between our and van de Voorde et al.’s [63] study is in the approach to productivity measurement and the level of analysis. While we applied a self-reported measure of productivity, they used yearly “branch profit per FTE index”. Additionally, we measured the psychological capital and they the aggregation of psychological climates—that is the organizational climate.

Our results also suggest that the relationship between perceived psychological climate for caring and certain work outcomes may be reciprocal. Given that we found the effect of climate for caring on work outcomes to be larger, we suspect that psychological climate can be an antecedent to these work outcomes, which is likely to later feed back into the climate of the organization. However, the sequence of these events in an organizational setting deserves further exploration.

As anticipated, our results confirm that a positive work environment leads to better work outcomes, reflecting the assumption that perceived psychological and organizational climates reveal employees’ views of how much an organization is concerned about their well-being and safety, thus influencing employee motivation and output [12,18,19,124]. Specifically, out of the four work outcomes we measured, three (self-reported productivity, work quality, and work engagement) were impacted by perception of the psychological climate for caring. This confirms hypothesis H1 for these three work outcomes but not for job satisfaction.

The effect of perceived psychological climate on productivity proved consistent with the findings of other scholars [42,53,55,125,126]. Our results on the effects of climate for caring on work quality are also aligned with the literature [127,128,129] (albeit mostly in the service industry and other fields that involve face-to-face customer interaction). They also show that psychological climate has an effect on subsequent work engagement, which corroborates findings from Brown and Leigh [67].

Our findings deviate from the literature on the effect of psychological climate and of ethical caring climate on job satisfaction [17,31,39], suggesting that some relationships seen in cross-sectional results may not hold in a longitudinal study with accounts for prior levels of outcomes and exposures (as also noted by Siehl and Martin [24]). Many cross-sectional studies show associations between psychological or organizational climates and job satisfaction [17,20,130,131], but we found no evidence of this relationship in the longitudinal setting with temporal cause and effect relationship between climate for caring and job satisfaction imposed by the data. One explanation is that determinants of job satisfaction might follow a hierarchy, with job characteristics, like promotional opportunity and task significance, as top predictors and organization characteristics, like psychological climate, as lesser predictors [132]. Other workplace resources, such as relationship with supervisors and coworkers, may also have more power in explaining overall job satisfaction [133,134,135]. Further, since our measure captures general job satisfaction, it is possible that psychological climate impacts certain aspects of job satisfaction, such as satisfaction with supervisors and managers, but not overall employee satisfaction with work [136]. Consequently, future research should consider more detailed instruments of job satisfaction, which would enable investigation of the effects of climate for caring on multiple job satisfaction facets and vice versa.

Our focus on a low- and semi-skilled workforce also adds to the literature, since earlier studies generally documented the psychological climate and work outcomes relationship in the service industry. Following the suggestion of Siehl and Martin [24], we focused on apparel factory workers, adding to the literature about perceptions of organizational climate and psychological climate of entry-level employees and not of top management only. It must be noted, however, that our sample of Mexican apparel workers may reflect specific job characteristics, worker characteristics, and cultural inclinations, which may constitute a limitation of the study. Different samples may yield different results as indicated by the international management literature. Differences in behavior, work values, and culture are acknowledged as ones of the biggest challenges for developing theories with cross-cultural relevance in the area of organizational studies [18]. However, some studies measuring the association between psychological climate and work outcomes using the same tools have been performed around the world, providing some evidence that the relationship may hold across cultures and geography [54].

Despite its strengths, our study has also certain limitations. While we used three waves of data, which let us control for a rich set of potentially confounding variables, demographic characteristics, outcomes prior to exposures and for prior values of exposure itself (i.e., strengthening the evidence for causality), the results may still be subject to unmeasured confounding, for example, personality. Likewise, work-related stress varies significantly across occupations [137,138], and therefore—although we controlled for job demand and job control, which are well known correlates of work-related stress and burnout [95,96]—one can expect this source of variation to affect the relationship between work outcomes and psychological climate. Next, our use of single-item instruments for job satisfaction, work quality, and work productivity is another limitation of the study. However, in the case of job satisfaction, our choice of a single-item global job satisfaction measure was supported by empirical studies showing satisfactory (and often superior) properties of the single-item instrument [76,77]. Additionally, attrition between waves may be a concern for the analyses. However, it was mostly due to the substantial turnover rates, confidentiality and data protection requirements in the studied factories that the number of workers surveyed at each of three occasions was rather low (approximately 35% of the original sample of Mexican workers participated in each subsequent wave of the survey). The follow-up period (i.e., one year) in this study might have also been an influencing factor. Shorter follow-up periods might be considered in future studies, especially in establishments with high turnover rates as the one examined by us. It might facilitate more complete understanding of short-term effects of the psychological climate for caring. It might also be that the one-year follow-up period was excessive to observe changes in the level of psychological climate for caring as well as its effects on work outcomes (and vice versa) especially in companies where the working conditions are demanding and remuneration unsatisfying. Shorter follow-up periods could have helped to collect data from newly employed workers who decided to quit job within the three-year period of the study. Finally, since our study uses self-reported data, it may be subject to social desirability bias [73]. In particular, self-reports of work quality and productivity may be subject to limited accuracy and reliability. However, the longitudinal character of the study provides a level of assurance that the findings are not entirely due to reporting bias.

Consequently, there should be caution as to the generalizability of our results, and more research for different job profiles and in different geographical contexts is needed to gain deeper insight. More research should be directed toward longitudinal (controlling for baseline exposure and for outcomes measured prior to exposure) rather than cross-sectional studies to improve understanding of the sequence of the causal relations between psychological climate and business outcomes, with special focus on antecedents of the climate. Shorter follow-up period should be considered as well as the examination of the impact of psychological climate on quitting job.

## 5. Conclusions

Our findings show that favorable perceptions of psychological climate (in our case: climate for caring) is beneficial for some work outcomes such as self-reported productivity, self-reported work quality, and work engagement. Therefore, this study provides empirical support for the non-recursive model of causal relationship between job perception domain of psychological climate and job satisfaction proposed by James and Tetrick [26] and the job demands-resources model by Bakker and Demerouti [27,28,29]. The findings also imply that in organizations with high self-reported productivity and work quality, beyond and above all other factors, it may be easier to create and establish a psychological climate for caring. Our study provides a pathway for action for leaders and managers who seek to improve organizational outcomes by creating and cultivating a work climate perceived as safe, caring, trusting, respectful, and fair. Specifically, managers could stimulate work engagement and improvement in performance and work quality by ensuring that employees feel safe, respected, and cared for. Furthermore, due to the feedback loops, managers might expect that fostering of psychological climate for caring in workplaces with high performance and work quality could bring about disproportionately larger effects than in workplaces with poor performance. The results of this study also convey a message to global brands and factory managers to foster both worker and organizational well-being, since they may improve factory performance.

## Figures and Tables

**Table 1:** Baseline characteristics of participants (at *T* = 1, *n* = 495).

Characteristic	%
Gender (women)	45.25
Age—mean (SD)	34.95 (10.06)
18–24	18.79
25–34	28.28
35–44	35.96
45 and older	16.97
Marital status (married)	60.46
Education (at least high school)	12.55
Having children under the age 18 currently living in the household	68.57
Being a primary caregiver for a parent or an elderly person currently living in the household	47.48
Job tenure	
Up to 1 year	3.03
More than 1 year and up to 5 years	34.75
More than 5 years	62.22

**Table 2:** Descriptive statistics and correlations between study variables.

Variable	Range	Mean (SD)	Correlations			
			(1)	(2)	(3)	(4)
T = 1						
Psychological climate for caring (1)	1–6	4.38 (1.27)	1			
Self-reported productivity (2)	1–6	4.45 (1.45)	0.69	1		
Self-reported work quality (3)	1–10	7.78 (2.55)	0.35	0.39	1	
Job satisfaction (4)	0–10	8.62 (2.16)	0.46	0.41	0.33	1
Work engagement (5)	0–6	4.38 (1.27)	0.60	0.54	0.36	0.53
T = 2						
Perception of organizational climate (1)	1–4	2.50 (0.80)	1			
Self-reported productivity (2)	1–4	3.01 (0.76)	0.44	1		
Self-reported work quality (3)	1–10	7.86 (2.32)	0.34	0.38	1	
Job satisfaction (4)	0–10	8.79 (1.82)	0.34	0.30	0.45	1
Work engagement (5)	0–6	4.92 (1.11)	0.43	0.33	0.30	0.62
T = 3						
Perception of organizational climate (1)	1–4	2.40 (0.79)	1			
Self-reported productivity (2)	1–4	2.93 (0.81)	0.46	1		
Self-reported work quality (3)	1–10	7.23 (2.43)	0.39	0.44	1	
Job satisfaction (4)	0–10	8.28 (2.22)	0.34	0.32	0.44	1
Work engagement (5)	0–6	4.65 (1.21)	0.37	0.42	0.41	0.64

All correlation coefficients are significant at 0.001 level.

**Table 3:** Standardized regression coefficients (standard errors in parenthesis) for the relationships between perception of psychological climate for caring and work outcomes (*n* = 495).

Work Outcome	Psychological Climate (*T* = 2) → Work Outcome (*T* = 3)	Work Outcome (*T* = 2) → Psychological Climate (*T* = 3)
Self-reported productivity	0.19 (0.05) t = 3.43; *p* = 0.001	0.17 (0.04) t = 3.84; *p* = 0.000
Self-reported work quality	0.14 (0.05) t = 2.65; *p* = 0.008	0.11 (0.05) t = 2.27; *p* = 0.024
Work engagement	0.16 (0.05) t = 3.39; *p* = 0.001	0.06 (0.06) t = 1.14; *p* = 0.255
Job satisfaction	0.07 (0.05) t = 1.36; *p* = 0.175	0.06 (0.05) t = 1.22; *p* = 0.225

All regression analyses were conducted controlling for: (1) demographic variables (gender, age, marital status, education, taking care for an elder, and having children below 18 at home); (2) job characteristics (job tenure, psychological and physical job demand, job control, learning opportunities, and physical working conditions); and (3) work-family conflict at *T* = 1. Additionally, (4) the first wave (*T* = 1) work outcomes and (5) first wave (*T* = 1) perception of organizational climate for caring were used as controls.
